# Anticancer activity mechanism of novelly synthesized and characterized benzofuran ring-linked 3-nitrophenyl chalcone derivative on colon cancer cells

**DOI:** 10.1515/med-2025-1310

**Published:** 2025-10-18

**Authors:** Melih Oztepe, Demet Coskun, Ferda Ari

**Affiliations:** Department of Biology, Faculty of Science and Arts, Bursa Uludag University, 16059, Bursa, Turkey; Department of Chemistry, Faculty of Science, Firat University, 23119, Elazig, Turkey

**Keywords:** apoptosis, cancer, chalcone, colon, cytotoxicity

## Abstract

Cancer is the second biggest cause of death after cardiovascular disorders and its incidence is rising significantly. One out of every ten cancer-related deaths is caused by colon cancer. The increasing incidence calls for creating focused therapeutic strategies with fewer adverse effects than traditional clinical techniques like radiation, chemotherapy, and immunotherapy. In this study, we evaluated the anticancer effects and mechanisms of a synthesized and characterized benzofuran ring-linked 3-nitrophenyl chalcone derivative, [1-(2-benzofuranyl)-3-(3-nitrophenyl)-2-propen-1-one], on colon cancer cells (HCT-116 and HT-29) as well as healthy colon cells (CCD-18Co). Cell viability analyses using the sulforhodamine B assay demonstrated that the IC₅₀ values after 48 h of treatment were 1.71 µM for HCT-116, 7.76 µM for HT-29, and higher than 10 µM for CCD-18Co cells. These results indicate a selective cytotoxic effect on cancer cells an essential criterion for evaluating anticancer compounds. Triple fluorescence staining, flow cytometry caspase 3/7 activity, along with protein expression analyses, confirmed that the compound induces apoptosis in both cancer cell lines. At IC₅₀ values, the derivative activated *DR-4*-mediated apoptosis at the membrane and *BCL-2*-mediated apoptosis intracellularly. Moreover, treatment with 12.5 µM of the compound for 24 h, corresponding to a cell cycle time, statistically significantly arrested the cell cycle at the G0/G1 phase. In addition, it inhibited cell migration and colony formation in a dose-dependent manner, starting from values as low as 1.56 µM. Additionally, the binding affinity of the derivative with target proteins was determined using artificial intelligence-assisted molecular modeling analysis. Collectively, these findings highlight the potential of this 3-nitrophenyl chalcone derivative as a promising candidate for the development of novel therapeutic agents against colon cancer.

## Graphical abstract

**Figure:**
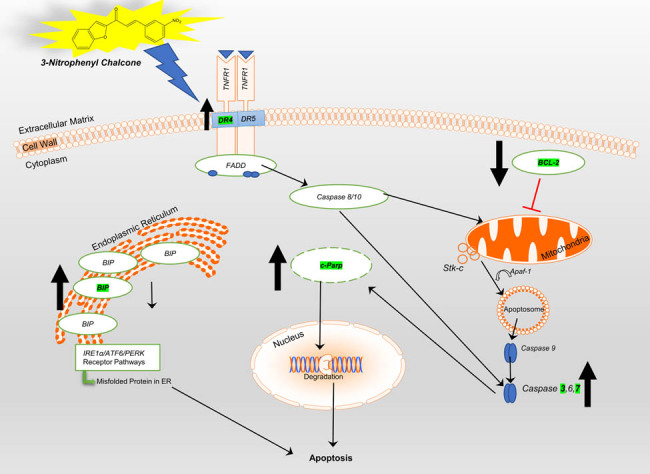

## 1 Introduction

One of the leading causes of death in the modern world is cancer, a disease marked by uncontrolled cell division brought on by genetic or epigenetic modifications and their dissemination to neighboring tissues [1]. According to the current data prepared by the International Agency for Research on Cancer worldwide, colorectal cancer, which accounts for 10% of cancer-related deaths, ranks third after breast and lung, respectively, in terms of incidence. In terms of mortality, it ranks second after the lung [2]. There are different approaches to cancer treatment (surgery, radiotherapy, immunotherapy, chemotherapy, etc.) [3,4,5,6]. Nevertheless, mortality rates are still high due to various reasons such as low stability and drug resistance [7]. Therefore, new treatment approaches with fewer undesirable side effects are needed than current approaches. When the homeostasis rate is disrupted in the cells of an adult organism, the pathway defined as apoptosis becomes active and physiological death occurs in an orderly manner [8]. Function or regulation disorders occurring in the apoptotic process are associated with various pathological conditions, especially cancer, autoimmune diseases, and the spread of viral infections [9]. In this regard, triggering the apoptotic death pathway is one of the primary goals of cancer treatment strategies.

The use of compounds synthesized from herbal or natural products in the treatment of cancer is very important considering that they have relatively fewer side effects compared to existing therapeutics [10]. Flavonoids, one of these compounds, have a polyphenolic structure and are found in pigment compounds in the plant [11]. Chalcones, from the flavonoid family, are compounds that can be found both naturally and synthetically in the laboratory [12]. It has been reported to affect biological activities such as anticancer activity, inhibition of free radical release in the body, chelating with metals [13]. Chalcone-derived chemicals have been shown to act on cancer cells through a variety of molecular processes, including apoptosis, DNA and mitochondrial damage induction, inhibition of angiogenesis, tubulin inhibition, kinase inhibition, and modifications in drug efflux protein activities [14,15].

**Figure 1:**
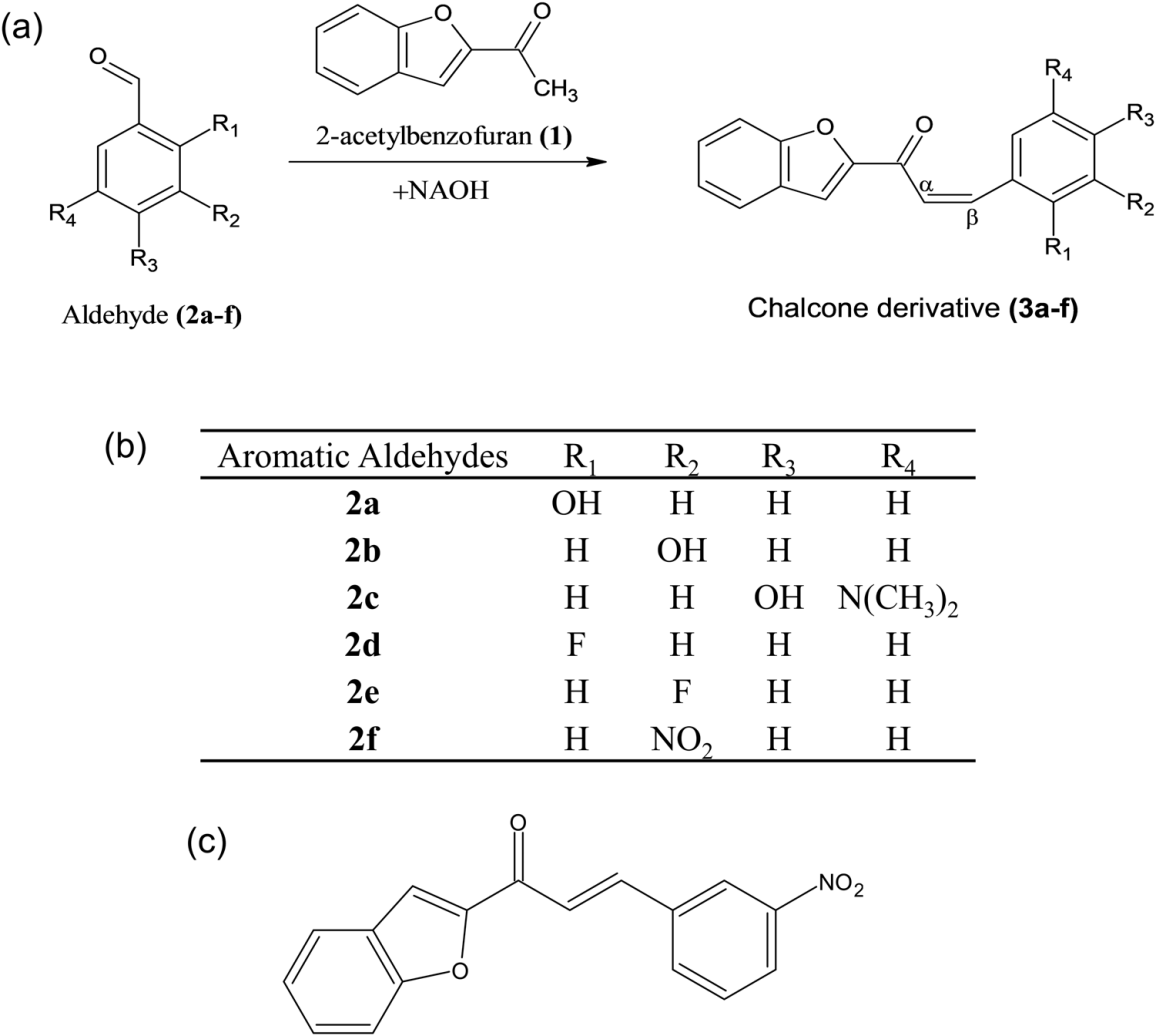
Synthesis reaction schemes of chalcone derivatives (a) containing different side groups (b). Molecular structure of 3-nitrophenyl chalcone derivative (c) (created with Chemsketch structure drawing software (https://www.acdlabs.com/products/chemsketch/, Research Resource Identifiers (RRID):SCR_019272)).

A review of the literature indicated that chalcone derivatives with different structural changes had anticancer potential. However, no research was found for the 3-nitrophenyl chalcone derivative (Figure 1c), whose synthesis and characterization are complete. Therefore, in this study, the anticancer effects and mechanisms of 3-nitrophenyl chalcone derivative on colon cancer cells (HCT-116 and HT-29) were revealed by *in-vitro* analyses.

## 2 Methods

### 2.1 Chemistry

#### 2.1.1 General

Solvents and reagents were bought from commercial suppliers and used according to their data sheets. We have produced chalcone derivatives by condensing different aromatic aldehydes with 2-acetylbenzofuran. First, a solution of 2-acetylbenzofuran (2.24 g, 14.0 mmol) and aldehyde (14.0 mmol) in methanol (25 mL) was cooled to 0–5°C. After that, this solution received 18 mL of water-soluble NaOH (1 mol/L) and was shaken for 3 h at room temperature. Overnight, the solution was let to stand in the refrigerator. After neutralization with HCl (%1 v/v), the solid precipitated upon the addition of water, and it was filtered and repeatedly rinsed with water. To obtain the chalcone, the solid substance was recrystallized using a suitable solvent.

Uncorrected melting points were determined with a Shimadzu DSC-50 differential scanning calorimeter. Elemental analyses were conducted using a Leco CHNS-932 device. KBr pellets were used to record infrared (IR) spectra using a Perkin-Elmer Spectrum One FTIR (Fourier transform infrared) spectrometer. Tetramethyl silane was used as the internal standard to determine ^1^H NMR spectra on a Bruker AC 300 (300 MHz) spectrometer and ^13^C NMR spectra were acquired on a Bruker (75.47 MHz) spectrometer. Chalcone derivatives were prepared at a stock value of 50 mM in dimethyl sulfoxide (DMSO). Following Baek’s procedure [16], 50 mM final DMSO value of derivatives was prepared to be 0.01(%) v/v. Fresh stock solutions were divided into 10 μL aliquots and stored in a −20°C freezer for use in further analyses.

#### 2.1.2 General procedure for the synthesis of chalcone derivatives

##### 2.1.2.1 3-(2-Hydroxyphenyl)-1-(benzofurane-2-yl)-2-propen-1-one (3a)

Yield: 2.03 g, 55%; mp193°C; FTIR (KBr, cm^−1^): 3,236, 3,000–3,100, 1,639, 1,614, 1,602, 1,575, 1,250, 1,141, 947, 764, 750; ^1^H NMR (300 MHz, CDCl_3_): *δ* 9.03 (s, 1H, OH), 8.08 (d, *J* = 8.37 Hz, 1H, b4-H), 7.72 (d, *J* = 7.20 Hz, 1H, b7-H), 7.22–7.68 (m, 9 H, all the others); ^13^C NMR: *δ* 179.68 (C =O), 155.90 (b8-C), 155.62 (b2-C), 152.53 (2-C), 141.91 (β-C), 132.01 (b9-C), 110.48–128.11 (the other Cs). Anal. calc. for C_17_H_12_O_3_: C, 77.29; H, 4.54. Found: C, 77.43; H, 4.35.

##### 2.1.2.2 3-(3-Hydroxyphenyl)-1-(benzofurane-2-yl)-2-propen-1-one (3b)

Yield: 2.15 g, 58%; mp 176°C; FTIR (KBr, cm^−1^): 3,226, 3,000–3,100, 1,639, 1,646, 1,612, 1,582, 1,229, 1,145, 973, 853, 785, 679; ^1^H NMR (300 MHz, DMSO-d_6_): *δ* 9.70 (broad, OH), 8.31 (s, 1H, b3-H), 7.87 (d, *J* = 7.76 Hz, 1H, b4-H), 7.82 (d, *J* = 15.69 Hz, 1H, β-H), 7.76 (d, *J* = 7.90 Hz, 1H, b7-H), 7.75 (d, *J* = 15.72 Hz, 1H, α-H), 7.57 (dd, *J* = 7.80 Hz and 7.36 Hz, 1H, b6-H), 7.40 (dd, *J* = 7.75 Hz and 7.40 Hz, 1H, b5-H), 6.91 (d, *J* = 7.20 Hz, 1H, 4-H), 7.27–7.33 (m, 3 H, 2,5, 6-H). Anal. calc. for C_17_H_12_O_3_: C, 77.29; H, 4.54. Found: C, 77.54; H, 4.54.

##### 2.1.2.3 3-(4-*N*,*N*-dimethylaminophenyl)-1-(benzofurane-2-yl)-2-propen-1-one (3c)

Yield: 3.26 g, 80%; mp 150°C; FTIR (KBr, cm^−1)^: 3,000–3,100, 1,643, 1,611, 1,569, 1,544, 1,522, 1,375, 1,359, 1,267, 1,152, 804, 746; ^1^H NMR (300 MHz, DMSO-d_6_): *δ* 8.16 (s, 1H, b3-H), 7.86 (d, *J* = 7.74 Hz, 1H, b4-C), 7.76 (d, *J* = 15.50 Hz, 1H, β-H), 7.75 (d, *J* = 6.80 Hz, 1H, b7-H), 7.73 (d, *J* = 8.83 Hz, 2 H, 2, 6-H), 7.60 (d, *J* = 15.51 Hz, 1H, α-H), 7.54 (dd, *J* = 7.80 and 7.03 Hz, 1H, b6-H), 7.39 (dd, *J* = 7.55 and 7.20 Hz, 1H, b5-H), 6.78 (d, *J* = 8.91 Hz, 2 H, 3, 5-H), 3.03 (s, 6 H, *N*,*N*-dimethyl). Anal. calc. for C_19_H_17_O_2_N: C, 78.37; H, 5.84; N, 4.81. Found: C, 77.93; H, 5.56; N, 4.42.

##### 2.1.2.4 3-(2-Fluorophenyl)-1-(benzofurane-2-yl)-2-propen-1-one (3d)

Yield: 2.02 g, 54%; mp 100°C; FTIR (KBr, cm^−1^): 3,120, 3,067, 1,659, 1,611, 1,576, 1,550, 1,292, 1,160, 753; ^1^H NMR (300 MHz, DMSO-d_6_): *δ* 8.30 (s, 1H, b3-H), 8.11 (dd, *J* = 7.60 and 7.20 Hz, 1H, b6-H), 7.93 (d, *J* = 15.87 Hz, 1H, β-H), 7.90 (dd, *J* = 8.37 and 7.55 Hz, 1H, b5-H), 7.89 (d, *J* = 15.87 Hz, 1H, α-H), 7.76 (d, *J* = 8.41 Hz, 1H, b4-H), 7.29–7.61 (m, 5 H, b7, 3, 4, 5, 6-H). Anal. calc. for C17H11O2F: C, 76.71; H, 4.13. Found: C, 76.45; H, 4.05.

##### 2.1.2.5 3-(3-Fluorophenyl)-1-(benzofurane-2-yl)-2-propen-1-one (3e)

Yield: 1.75 g, 47%; mp 99°C; FTIR (KBr, cm^−1^): 3,118, 3,073, 1,662, 1,611, 1,582, 1,545, 1,278, 1,156, 981, 850, 753; ^1^H NMR (300 MHz, DMSO-d6): *δ* 8.39 (s, 1H, b3-H), 7.97 (d, *J* = 15.60 Hz, 1H, β-H), 7.70–7.90 (m, 3 H, b4, b6, α-H), 7.35–7.63 (m, 6 H, b5, b6, 2, 4, 5, 6-H). Anal. calc. for C17H11O2F: C, 76.71; H, 4.13. Found: C, 76.57; H, 4.18.

##### 2.1.2.6 3-(3-Nitrophenyl)-1-(benzofurane-2-yl)-2-propen-1-one (3f)

Yield: 2.13 g, 52%; mp 189°C; FTIR (KBr, cm^−1^): 3,000–3,120, 1,669, 1,615, 1,549, 1,521, 1,358, 1,282, 1,165, 1,040, 976, 812, 740; ^1^H NMR (300 MHz, DMSO-d6): *δ* 8.79 (s, 1H, 2-H), 8.43 (s, 1H, b3-H), 8.33 (d, *J* = 7.74 Hz, 1H, 4-H), 8.27 (dd, *J* = 7.41 and 1.52 Hz, 1H, 6-H), 8.08 (d, *J* = 15.77 Hz, 1H, β-H), 7.92 (d, *J* = 15.90 Hz, 1H, α-H), 7.90 (d, *J* = 7.50 Hz, 1H, b4-H), 7.74–7.79 (m, 2 H, 5, b7-H), 7.58 (dd, *J* = 7.60 and 7.62 Hz, 1H, b6-H), 7.40 (dd, *J* = 7.51 and 7.48 Hz, 1H, b5-H). Anal. calc. for C17H11O4N: C, 69.64; H, 3.75; N, 4.78. Found: C, 70.03; H, 3.59; N, 4.65.

### 2.2 Pharmacology/Biology

#### 2.2.1 Cell lines

HCT-116 and HT-29 colon cancer cells were cultivated in Roswell Park Memorial Institute 1640 (including L-glutamine) medium that was enhanced with 1% non-essential amino acid, penicillin G (100 U/mL), streptomycin (100 μg/mL), and 10% fetal bovine serum. Healthy colon cells CCD-18Co cells were cultivated in Dulbecco’s Modified Eagle Medium (including L-glutamine) with additional supplements of 10% fetal bovine serum, penicillin G (100 U/mL), and streptomycin (100 μg/mL). All cells have been attained from American Type Cell Culture Collection (ATCC, USA) and cultured in incubator at 37℃ with 5% CO_2_ throughout the analyses.

#### 2.2.2 Detection of cell cytotoxicity by sulforhodamine B (SRB) viability analysis

SRB is a bright-purple amino xanthene stain with sulfonic groups that bind to basic amino acid side chains below acidic situations and dissociate below basic situations. The method allows quantitative analysis of cell viability as a result of reading the SRB dye [17]. To ascertain the potential cytotoxic effect, 3 replicates of HCT-116, HT-29, and CCD-18Co cells were first added to 96-well plate at 5 × 10^3^ cells/well in 100 μL. It was incubated overnight to ensure that the cells adhered to the plate surface. To obtain preliminary data, 6 chalcone derivatives (3a–f) (0.78–50 μM) were added to the experimental group. Additionally, viability analysis was performed similarly to determine the possible effect of the solvent (DMSO) of the derivative. Following 24 and 48 h treatment, SRB viability analysis was carried out as previously mentioned [18]. At the end of the treatment periods, the cells were fixed to the plate bottom with 50% (w/v) trichloroacetic acid for 1 h at 4℃. After the plate was washed five times with distilled water, it was incubated with 0.4% (w/v) SRB for 30 min at 37℃. To remove unbound dye, the wells were washed five times with 1% (v/v) acetic acid solution. 10 mM Tris base (pH: 10.0) was added to the wells to solubilize the dye bound to the fixed cells. To measure the percentage of viability spectrophotometrically, absorbance (Abs) was measured at a wavelength of 564 nm on the Elisa Reader. As a result of SRB analysis, the most effective derivative was selected and further analysis was performed. Viability rates (%) in cells were calculated using the formula below. Subsequently, IC_50_ (Half-maximal inhibitory value) values were calculated according to the viability (%) results. IC_50_ values were determined by normalizing each cell group to the groups without chalcone derivative (negative control).

\[{\mathrm{Viability}}( \% )=100\times \mbox{''}{\mathrm{Abs\; value\; of\; experimental\; group}}/{\mathrm{Abs\; value\; of\; control\; group}}\mbox{''}]\]

To more comprehensively evaluate the antiproliferative activity of the compound, the ratio of IC_50_ of healthy cells to cancerous cells must be determined. The Selectivity Index (SI) values were computed for this purpose using the following formula [19]:

\[{\mathrm{SI}}={{\mathrm{IC}}}_{50}{\mathrm{value\; of\; healthy\; cells}}/{{\mathrm{IC}}}_{50}{\mathrm{value\; of\; cancer\; cells}}]\]

#### 2.2.3 Detection of cell death pathway

Fluorescent dyes generally bind to different compartments of the cell and provide morphological status information [20,21]. To determine the state of the cell in the apoptotic and necrotic death pathway, first, HCT-116 and HT-29 cells were added to 96-well plate at 5 × 10^3^ cells/well in 100 μL and three replicates. It was incubated overnight to ensure that the cells adhered to the plate surface. 3-nitrophenyl chalcone derivative (3.125–25 μM) was added to the experimental group. Following 24 h treatment, staining was carried out using the previously mentioned dyes Hoechst 33,342 (4 μg/mL), Annexin-V (AnxV; 3 μg/mL), and Propidium Iodide (PI; 1 μg/mL) [22] and examined under a fluorescent microscope. The densitometric analysis was carried out with Image-J (https://imagej.net/ij/, RRID:SCR_003070). After normalization, the stages of apoptosis in the cells were determined.

Cell death is regulated by caspases involved in the apoptosis [23]. To determine caspase 3/7 activity, first, HCT-116 and HT-29 cells were added to 6-well plate at 1 × 10^5^ cells/well in 1 mL and three replicates. It was incubated overnight to ensure that the cells adhered to the plate surface. 3-nitrophenyl chalcone derivative (3.125–12.5 μM) was added to the experimental group. Following 48 h treatment, analysis was carried out at the Muse Cell Analyzer (Millipore, Germany) using the caspase 3/7 kit (Luminex, USA) in compliance with the manufacturer’s instructions.

Western blotting technique enables the detection of proteins, which is the last step of the genetic information flow. In brief, it relies on the migration of proteins in a polyacrylamide gel through an electrophoresis process to a support membrane, with the proteins in the membrane being measured using immunological techniques [24]. First, HCT-116 and HT-29 cells were added to 6-well plate at 1 × 10^5^ cells/well in 1 mL volume and three replicates. It was incubated overnight to ensure that the cells adhered to the plate surface. 3-nitrophenyl chalcone derivative (1.56–12.5 μM) was added to the experimental group. Following 48 h treatment, cell lysate preparation and western blot analysis were carried out as previously mentioned [25]. Western blot was performed using *GAPDH* (Glyceraldehyde 3‐phosphate dehydrogenase; Cat# 5174; RRID: AB_10622025), *PARP* (Poly (ADP‐ribose) polymerase 1; Cat# 9542, RRID: AB_2160739), *c-PARP* (cleaved *PARP*, Large fragment; Cat# 9546; RRID: AB_2160593) *BIP* (Binding-immunoglobulin protein; Cat# 3177; RRID: AB_2119845), *DR-4* (Death Receptor 4; Cat# 42533; RRID: AB_2799223), and *BCL-2* (B‐cell lymphoma 2; Cat# 2876; RRID: AB_2064177) primary antibodies (Cell Signaling Technology). The HRP-linked anti-rabbit Ig (Cat# 7074; RRID: AB_2099233) seconder antibody (Cell Signaling Technology) was then used for the second antibody incubation. The Image Quant LAS 4000 (GE Healthcare 9410 Variable Mode Imager; RRID:SCR_018047) was utilized to visualize bound antibodies. The densitometric analysis was carried out with Image-J. Blot images of both cells normalized according to *GAPDH* and compared to control, which is set to 1.0.

#### 2.2.4 Analysis based on measurement of growth and proliferation abilities

As a result of possible mutations or changes, cells escape from the Cyclin-Dependent Kinase (*CDK*) proteins that control cellular proliferation and enter the process of carcinogenesis as they proliferate excessively. Over time, these cells divide uncontrollably, becoming more resistant to tumor suppressor proteins such as *p53* that protect healthy tissue, and become less dependent on signals from other cells [26]. Therefore, targeting the cell cycle is one of the main goals in cancer treatment. First, HCT-116 and HT-29 cells were added to 6-well plate at 1 × 10^5^ cells/well in 1 mL and three replicates. To enable the cells adhesive to the plate surface, it was incubated for the entire night. 3-nitrophenyl chalcone derivative (3.125–12.5 μM) was added to the experimental group. The cells were taken off the plate surface after 24 h, and their lysates were extracted in 70% cold ethanol. The “Muse^®^ Cell Cycle Reagent” on the Muse Cell Analyzer (Millipore, Germany) was used to determine the distribution of cells in the cycle phases following the Muse Cell Cycle Kit (Luminex, USA) process.

Monolayer wound healing analysis was performed to determine the cell migration rate. First, HCT-116 and HT-29 cells were added to 6-well plate at 15 × 10^4^ cells/well in 1 mL and three replicates. Using 200 μL pipette tip, horizontal and vertical lines were drawn on each well surface once the cells had reached 90% occupancy (confluent condition) and 3-nitrophenyl chalcone derivative (1.56–12.5 μM) was added to the experimental group. The moment of wound creation was considered as the beginning and healing was recorded from the same point at regular intervals throughout the 48 h treatment. The densitometric analysis was carried out with Image-J. Migration rate normalized and compared to 0th h, which is set to 0(%) [27,28].

Using this technique, the population’s capacity for “unlimited” division is assessed for every cell [29,30]. First, HCT-116 and HT-29 cells were added to 6-well plate at 2,000 cells/well in 1 mL and three replicates. Since the cells were seeded in relatively small amounts, they were incubated overnight to ensure adhesion to the plate surface. 3-nitrophenyl chalcone derivative (1.56–6.25 μM) was added to the experimental group. The derivative was taken out of the well at the end of the 48 h and fresh medium was applied. The media in the wells were replaced with fresh media every 3 days until at least 50 cells per colony were formed in the control group cells, and the growth of the cells was examined under an inverted microscope at regular intervals. After a sufficient density of colonies was formed in the control group cells, the medium in the wells was removed, 1 mL of cold methanol was added to each well and fixed at −20°C for 15 min. To remove methanol, the wells were washed once with 1 mL Phosphate buffer saline (PBS, 1×, Sigma-Aldrich). Then, they were stained with 1 mL of 0.05% crystal violet (prepared in methanol) by incubating them in a dark environment at room conditions for 20 min. At the end of the time, it was washed once again with 1 mL PBS (1×) and images of the cells were recorded. The densitometric analysis was carried out with Image-J. Colony formation rate was compared to control, which is set to 100(%).

### 2.3 Molecular docking analysis

The data file (.pdb format) for the 3D model of the 3-nitrophenyl chalcone derivative used in this study was generated from the SMILES representation using the RDKit library in Python and energy optimization was performed. Then, 3D structures of DR-4 and BCL-2 proteins (.pdb format), which were predicted to be associated with the 3-nitrophenyl chalcone derivative, were obtained using a similar method [31].

In the second stage, molecular docking interaction analysis was conducted using the CB-Dock2 (Cavity-detection guided Blind Docking) protein-ligand interaction database [32]. AutoDock Vina scores (kcal/mol), obtained from docking analysis performed in the CB-Dock2 system, estimate how strongly a compound interacts with its target protein. Each protein-ligand molecule has a CurPocket ID number, defined according to its interaction pattern at different coordinates in the 3D model. In this context, the interaction type with the lowest negative level has the potential to create strong bonds from a chemoinformatical perspective [33].

### 2.4 Statistical data analysis

The mean of at least three separate investigations was calculated to display the experiment outcomes with ± standard deviation (SD). GraphPad Prism v9.2.0 (RRID:SCR_002798) was used to evaluate all statistical analyses and one-way analysis of variance was used for testing. Values of *p < 0.05; **p < 0.01; and ***p < 0.001 were deemed statistically significant [34].

## 3 Results

### 3.1 Chemistry

For biological activity analyses, six chalcone derivatives with different side groups were re-synthesized following the method previously synthesized and characterized by our group [35]. Chalcone derivatives with the 3-(substituted aryl)-1-benzofuranyl-2-propenone skeleton (Figure 1a and b) were obtained by condensation of 2-acetylbenzofuran(1) and six aromatic aldehydes (2a–f). These aldehyde substrates are 2-hydroxybenzaldehyde, 3-hydroxybenzaldehyde, 4-(dimethylamino) benzaldehyde, 2-fluorobenzaldehyde, 3-fluorobenzaldehyde, 2-nitrobenzaldehyde, respectively. The most common method for the synthesis of chalcones is the Claisen–Schmidt reaction, which involves the condensation of a benzaldehyde derivative with an acetophenone derivative in ethanol solvent using sodium hydroxide [36]. All chalcones were purified by recrystallization from ethanol. All derivatives were characterized by elemental analysis, Fourier transform infrared (FT-IR), ^1^H NMR, and ^13^C NMR (only 3a). Derivatives showed characteristic bands between 1,639 and 1,662 cm^−1^ (C═O stretching) and 1,605 and 1,615 cm^−1^ (C═C stretching) at wavelengths varying according to the structure. In ^1^H NMR spectra, the most characteristic bands were observed at 8.08–8.43 ppm (3b–h) and 7.60–8.00 ppm (α-H and β-H, the resonance of β-H is in a lower area than α-H) and a coupling constant of about 15 Hz characterized the trans-configuration.

### 3.2 Pharmacology/biology

#### 3.2.1 3-nitrophenyl chalcone derivative selectively reduces the viability of cancer cells

**Figure 2:**
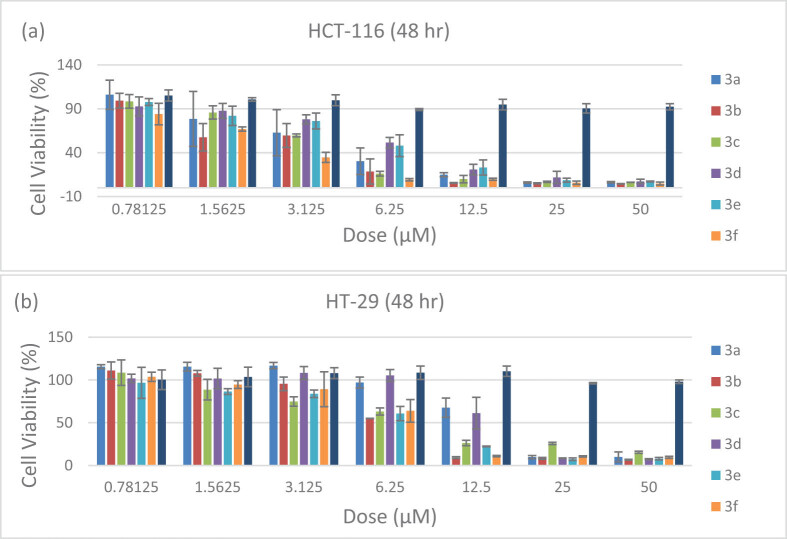
Line diagrams of SRB analysis results after treatment with six chalcone derivatives on HCT-116 (a) and HT-29 (b) cells for 48 h.

First, six types of chalcone derivatives (3a–f) were applied to colon cancer (HCT-116 and HT-29) cells with different genomic and morphological features for 48 h and the most effective derivative was determined as 3-nitrophenyl chalcone (3f) as a result of SRB viability analysis (IC_50_ 2.38; 7.89, respectively) (Figure 2). It was observed that the solvent of the derivative (DMSO) did not affect cancer cells, which would reduce their viability.

**Figure 3:**
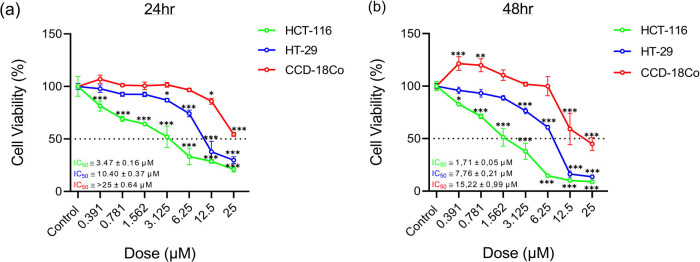
Line diagrams of SRB analysis results after treatment with 3-nitrophenyl chalcone derivative (0.391–25 μM) on HCT-116, HT-29, and CCD-18Co cells for 24 h (a) and 48 h (b). * Indicates statistically significant differences compared to the control (*p < 0.05; **p < 0.01; ***p < 0.001). Data are shown as mean value ± SD (*n* = 3).

Then, viability analysis was performed for 24 h (Figure 3a) and 48 h (Figure 3b) to determine the possible selective effect between healthy (CCD-18Co) and cancer (HCT-116 and HT-29) cells. It was observed that there was a general decrease in the viability percentages (%) in cancer cells (HCT-116 and HT-29) as the dose increased at both times, and that it was statistically significantly (*p < 0.05; ***p < 0.001) lower in HCT-116 cells, especially at low doses (0.391–6.25 μM). Depending on the change in treatment (from 24 to 48 h), it was revealed that cytotoxicity increased more in HCT-116 cells (51%) than in HT-29 cells (25%). It was observed that the viability of healthy cells was higher than cancer cells, even though it decreased depending on time and dose. It was determined that cytotoxicity was very low (>25 μM) at the 24 h, and viability was decreased by half at the 15.22 μM dose at 48 h. Additionally, the formulated SI value was calculated to determine the cell-specific selectivity of the derivative. According to the results, it was concluded that the SI value increased in HCT-116 cells (from 8.18 to 8.9 μM) and decreased in HT-29 cells (from 2.73 to 1.96 μM) depending on time (Table 1).

**Table 1:** SI values of 3-nitrophenyl chalcone derivative on HCT-116 and HT-29 cells according to the SRB analysis results obtained after 24 h (a) and 48 h (b) treatment

SI	24 h		48 h	
	HCT-116	HT-29	HCT-116	HT-29
3-nitrophenyl chalcone derivative	8.18	2.73	8.9	1.96

#### 3.2.2 3-nitrophenyl chalcone derivative promotes apoptotic cell death

**Figure 4:**
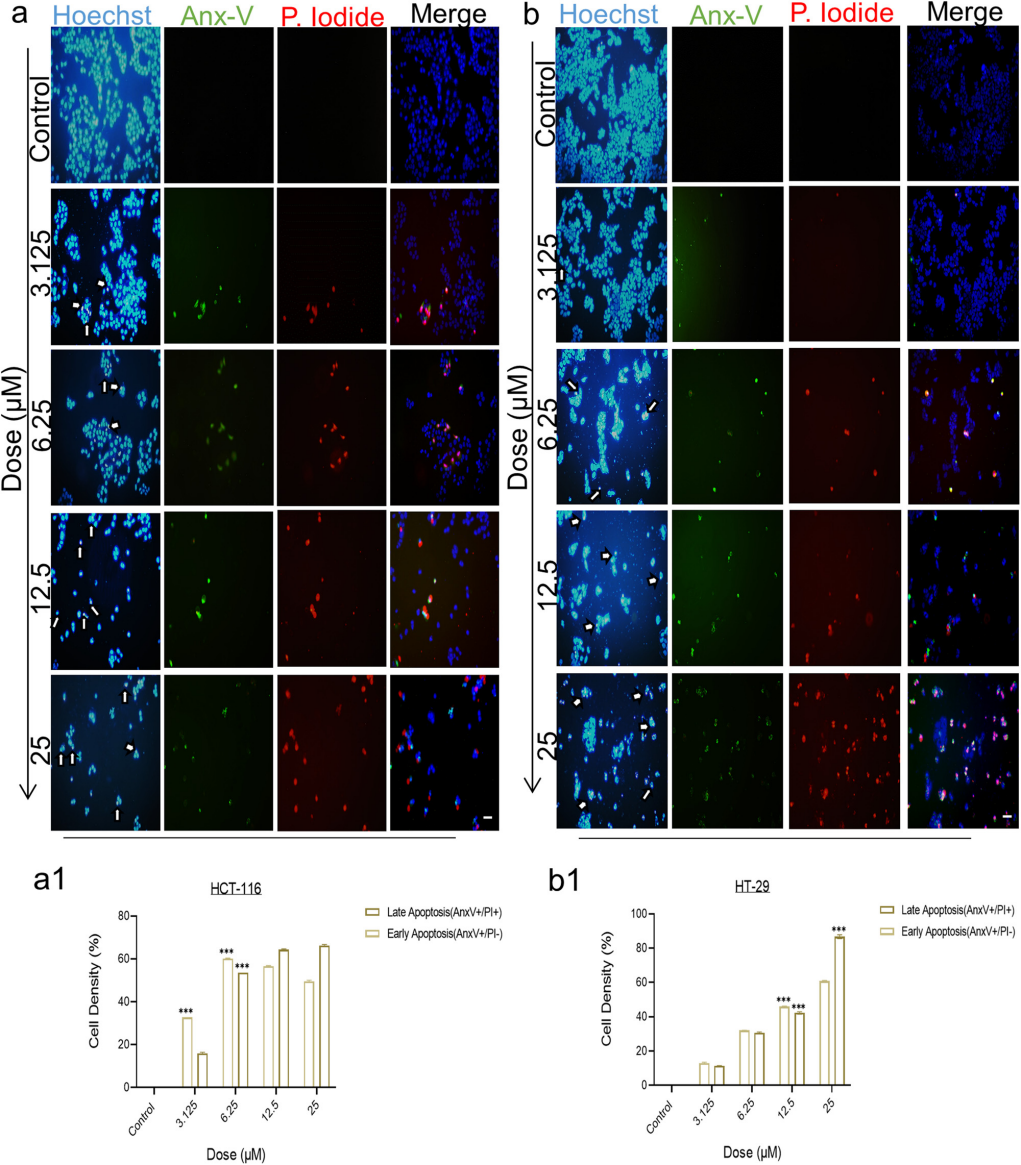
Fluorescent staining images of Hoechst (blue labeled), Annexin V (green labeled), and Propidium Iodide (red labeled) after treatment with 3-nitrophenyl chalcone derivative (3.125–25 μM) on HCT-116 (a) and HT-29 (b) cells for 24 h. White arrows indicate Pyknotic nuclei. The histogram graphs express the density (%) and late apoptotic cells on HCT-116 (a1) and HT-29 (b1) cells according to the densitometric data. *Indicates statistically significant differences compared to the control (***p < 0.001). Data are shown as mean value ± SD (*n* = 3). Objective magnification ×20 and the scale bar length 50 μm.

To detect the death mode, HCT-116 and HT-29 cells were subjected to 3-nitrophenyl chalcone derivative (3.125–25 μM, determined according to the viability analysis results) for 24 h. According to the results, in HCT-116 (Figure 4a) and HT-29 (Figure 4b) cells decrease in the density of Hoechst + cells and increase in the density of apoptosis-specific pyknotic cells were generally observed as the dose increased. In HCT-116 (Figure 4a1) cells, it was revealed that early apoptotic (AnxV+/PI−) cells were statistically significantly (***p < 0.001) more dense at low doses (3.125 and 6.25 μM), while late apoptotic (AnxV+/PI+) cells were more dense at higher doses (12.5 and 25 μM). In HT-29 (Figure 4b1) cells, it was revealed that the cell density in both stages increased at similar rate as the dose increased. At the 25 μM dose, unlike other doses, late apoptotic (AnxV+/PI+) cells were statistically significantly (***p < 0.001) more dense. It was seen that the fluorescence microscopy results in both cells supported our viability results.

**Figure 5:**
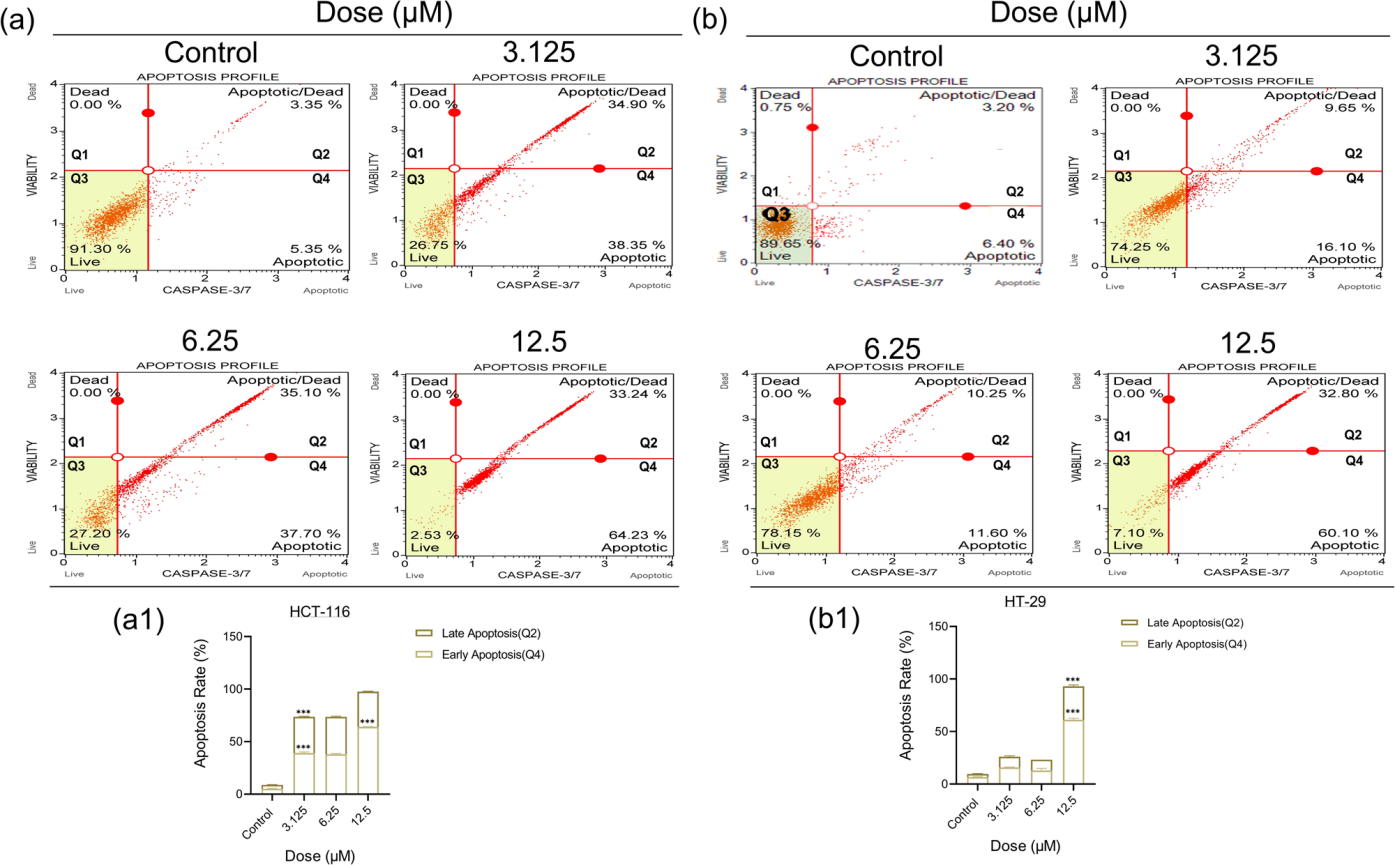
Results of Caspase-3/7 activation analysis using flow cytometry after treatment with 3-nitrophenyl chalcone derivative (3.125–12.5 μM) on HCT-116 (a) and HT-29 (b) cells for 48 h. The histogram graphs express the percentage (%) early (Q4), late (Q2), and total (Q2 + Q4) apoptotic cells according to the quantitative data of HCT-116 (a1) and HT-29 (b1) cells, respectively. *Indicates statistically significant differences compared to the control (***p < 0.001). Data are shown as mean value ± SD (*n* = 3).

To determine the level of Caspase 3/7, which is involved in the death through apoptosis, HCT-116 and HT-29 cells were subjected to 3-nitrophenyl chalcone derivative (3.125–12.5 μM, determined according to the viability analysis results) for 48 h. According to the results, it was observed that the apoptotic rate in HCT-116 (Figure 5a) cells increased statistically significantly (***p < 0.001) starting from low doses (3.125 μM) and approached the maximum value at the dose of 12.5 μM. Moreover, it (Figure 5a1) was revealed that death due to dose increase generally occurred in the early stage of apoptosis (Q4). Although the increase in apoptotic rate was observed to be low (<⁓22%) in HT-29 (Figure 5b) cells at doses of 3.125 and 6.25 μM, it was determined to be statistically significantly (***p < 0.001) higher (⁓93%) at the dose of 12.5 μM. Similar to HCT-116 cells, it (Figure 5b1) was revealed that death due to dose increase generally occurred in the early stage of apoptosis (Q4). It was seen that the Caspase 3/7 results in both cells supported our viability results.

**Figure 6:**
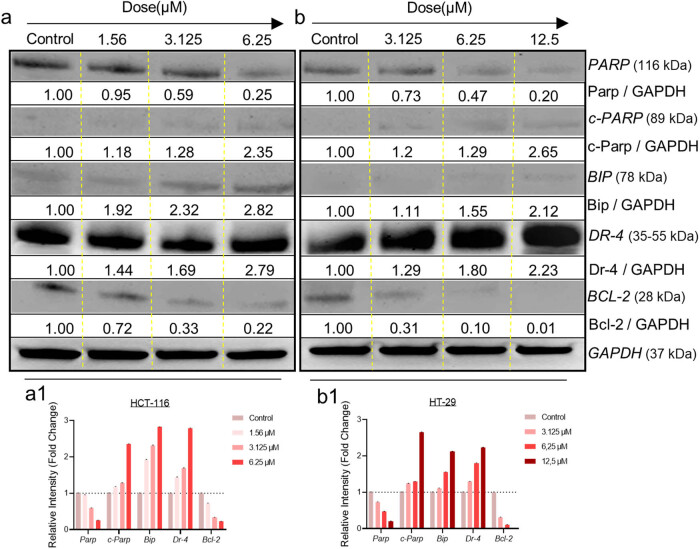
Blot images and fold change values of some apoptosis pathway proteins acquired with western blot analysis after treatment with 3-nitrophenyl chalcone derivative (1.56–12.5 μM) on HCT-116 (a) and HT-29 (b) cells for 48 h. Equal protein loading confirmed by *GAPDH*. The histogram graphs express the relative intensity of some apoptosis pathway proteins on HCT-116 (a1) and HT-29 (b1) cells, respectively.

To measure the amounts of certain proteins involved in apoptosis-induced death, HCT-116 and HT-29 cells were subjected to 3-nitrophenyl chalcone derivative (1.56–12.5 μM, determined according to the viability analysis results) for 48 h. The results are shown from top to bottom according to the kDa values of the proteins (Figure 6a and b). In both cells, *PARP* decreased and *c-PARP* increased depending on the dose increase. *BIP* increased significantly in both cells starting from the dose of 1.56 μM (Figure 6a1) and 6.25 μM (Figure 6b1), respectively. Similarly, an increase at *DR-4* was observed in both cells. Finally, it was observed that “anti-apoptotic” *BCL-2*, which plays a role in the intrinsic pathway of apoptosis, decreased more in HT-29 cells at the same dose values. In parallel with our viability and caspase 3/7 activity results, it was concluded that the derivative was more effective in HCT-116 cells when compared to western blot results at the same dose values.

#### 3.2.3 Analyses based on measurement of growth and proliferation abilities

**Figure 7:**
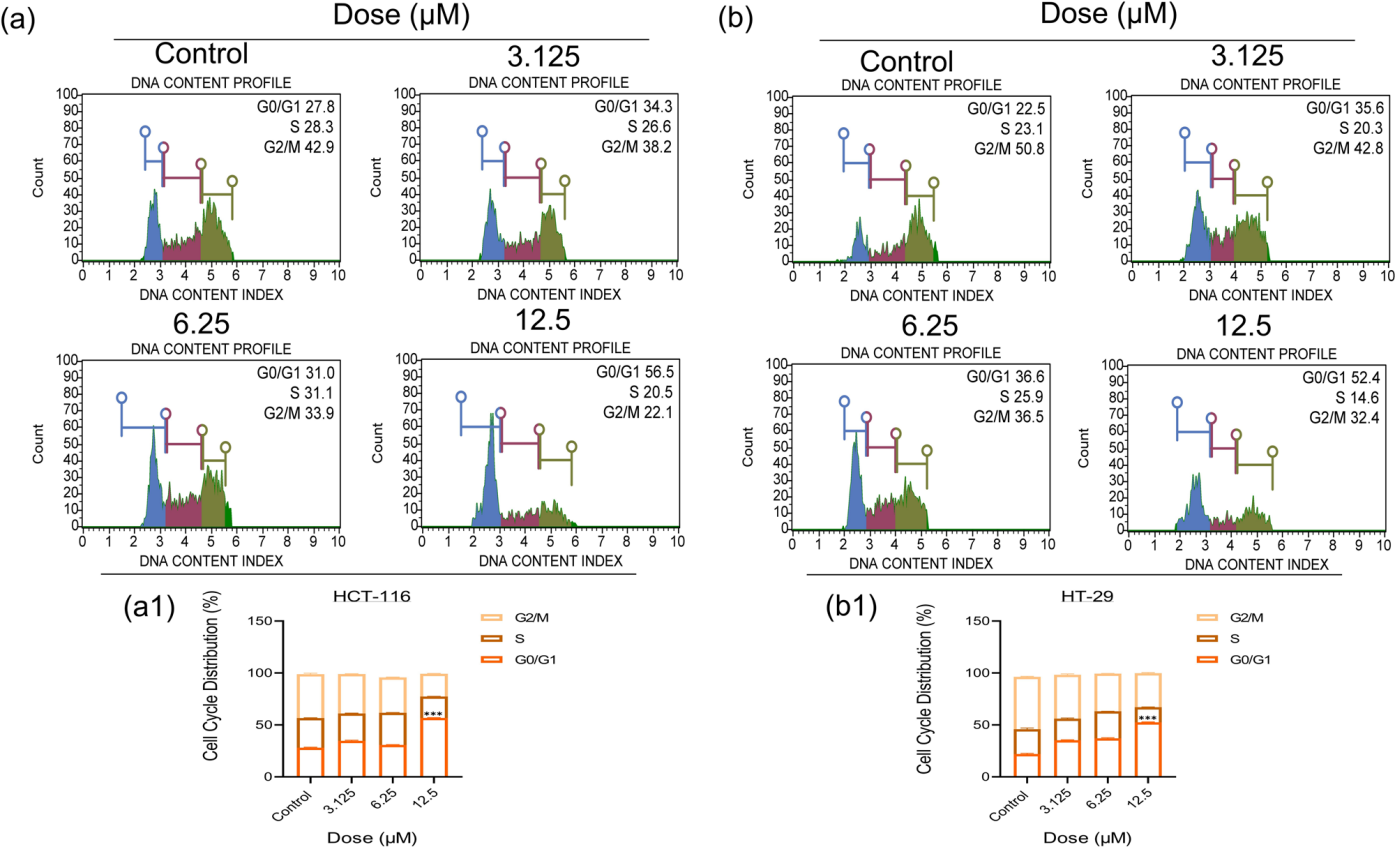
Results of cell cycle analysis using flow cytometry after treatment with 3-nitrophenyl chalcone derivative (3.125–12.5 μM) on HCT-116 (a) and HT-29 (b) cells for 24 h. The histogram graphs express the distribution (%) of cell cycle stages according to the quantitative data of HCT-116 (a1) and HT-29 (b1) cells, respectively. *Indicates statistically significant differences compared to the control (***p < 0.001). Data are shown as mean value ± SD (*n* = 3).

To detect the stage at which division is suppressed, HCT-116 and HT-29 cells were subjected to 3-nitrophenyl chalcone derivative (3.125–12.5 μM, determined according to viability analysis results) for 24 h. According to the results, the cell arresting rate in HCT-116 (Figure 7a) cells increased and decreased starting from 3.125 μM dose in the G0-G1 phase, and finally increased to 56.5% at the 12.5 μM dose, resulting in a statistically significant (***p < 0.001) increase. It was concluded that the cycle was suppressed before entering the DNA synthesis phase (Figure 7a1). In the S phase, it was observed that the arresting rate decreased and increased depending on the increase in dose, and it was determined that there was 20.5% arrest rate at 12.5 μM.

It was concluded that in the G2/M phase, which is the preparation phase for entry into mitosis, a cell arresting rate of 42.9% was observed in the control group, while the arresting rate decreased steadily depending on the increase in dose. In HT-29 (Figure 7b) cells, the cell arresting rate increased statistically significantly (***p < 0.001) from 22.5 to 52.4% depending on the dose increase in the G0-G1 phase, and it was concluded that the cycle was suppressed (Figure 7b1). While there was 23% arresting rate in control group cells in S phase, it decreased and increased depending on the increase in dose, and it was observed that it decreased to 14.6% at a dose of 12.5 μM. It was concluded that in the G2/M phase, while 50.8% cell arresting was observed in the control group, the rate decreased regularly depending on the increase in dose. Cell cycle results support the anticancer activity of the derivative observed in our other analyses.

**Figure 8:**
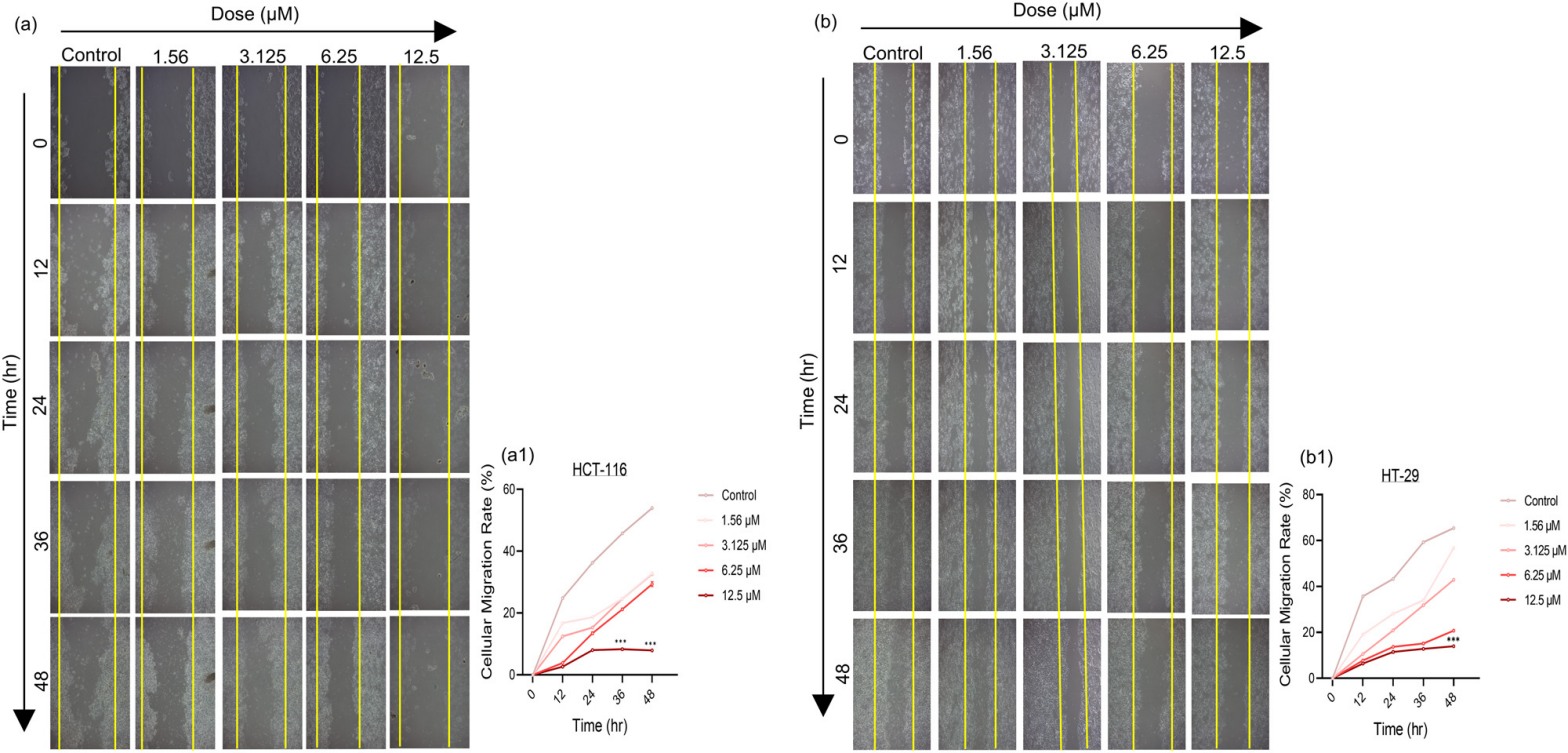
Results of wound healing analysis after treatment with 3-nitrophenyl chalcone derivative (1.56–12.5 μM) on HCT-116 (a) and HT-29 (b) cells up to 48th h. The line graphs express the cellular migration rate (%) of HCT-116 (a1) and HT-29 (b1) cells within the yellow vertical bar according to the densitometric data. *Indicates statistically significant differences compared to the control (***p < 0.001). Data are shown as mean value ± SD (*n* = 3). Objective magnification ×4.

To observe the ability to migration, HCT-116 and HT-29 cells were subjected to 3-nitrophenyl chalcone derivative (1.56–12.5 μM, determined based on viability analysis results) for 48 h. According to the results, it was observed that migration in HCT-116 (Figure 8a) and HT-29 (Figure 8b) cells generally decreased with increasing dose. When comparing same doses of the derivative, it was revealed that there was more effect at low doses (1.56 and 3.125 μM) in HCT-116 (Figure 8a1) cells, while there was more effect at high doses (6.25 and 12.5 μM) in HT-29 (Figure 8b1) cells. In this context, the dose values that affected both cells in the migration results were found to be compatible with the cytotoxic dose values determined in our 48-h viability results. Additionally, when the migration rates at the 48th h were compared, it was observed that it was statistically significantly (***p < 0.001) lower (⁓10%) at the 12.5 μM dose in both cells. Yellow bars indicate the area examined in the statistical analysis of the migration ability of cells.

**Figure 9:**
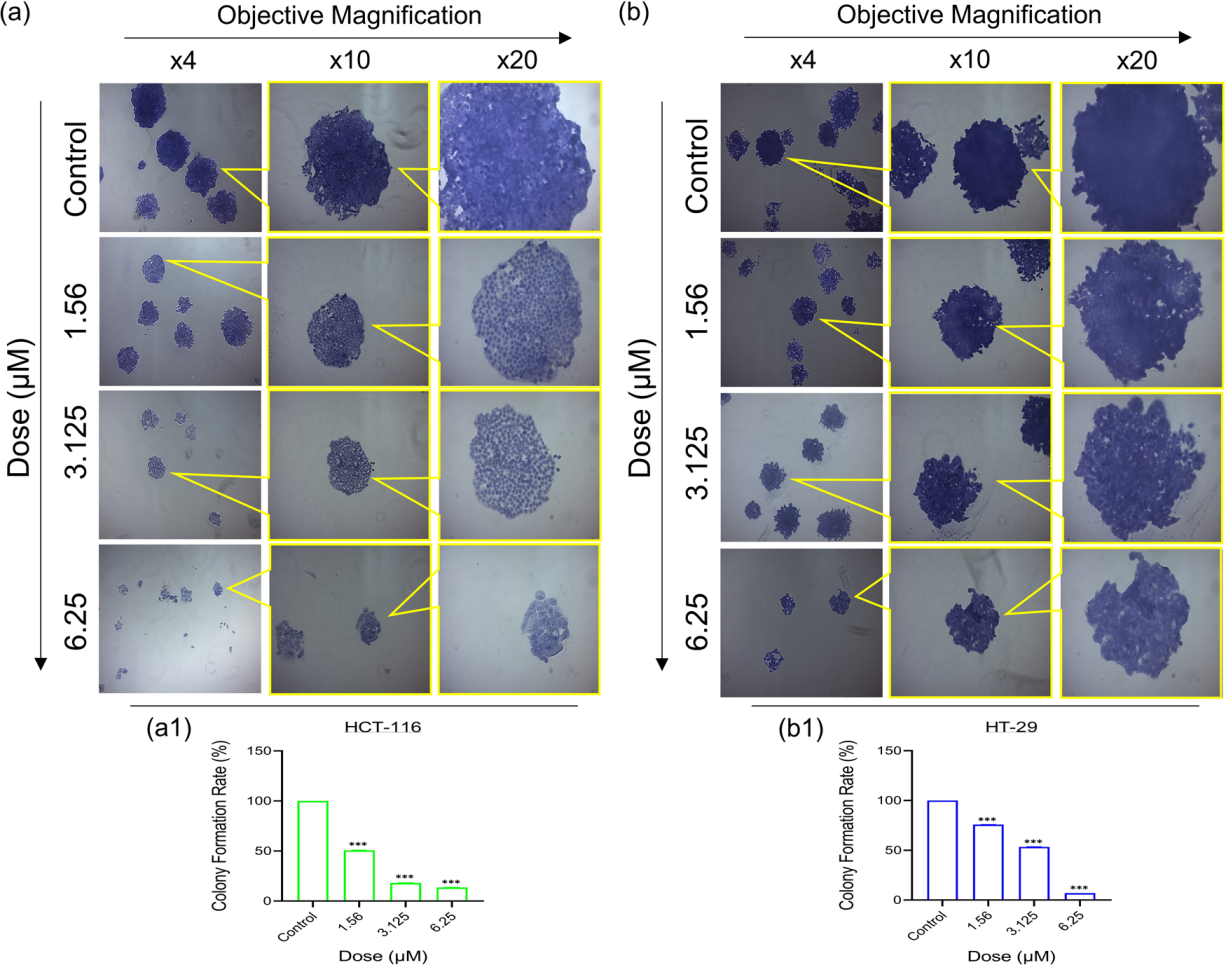
Results of colony formation analysis after treatment of HCT-116 (a) and HT-29 (b) with 3-nitrophenyl chalcone derivates (1.56–6.25 μM) for 48 h. The histogram graphs express the colony formation rate (%) of HCT-116 (a1) and HT-29 (b1) cells, respectively, according to the densitometric data of ×4 objective magnification. *Indicates statistically significant differences compared to the control (***p < 0.001). Data are shown as mean value ± SD (*n* = 3).

To ascertain whether every cell in the surroundings is capable of “unlimited” division, HCT-116 and HT-29 cells were subjected to 3-nitrophenyl chalcone derivative (1.56–12.5 μM, determined based on viability analysis results) for 48 h. According to the results, it was observed that there were statistically significant (***p < 0.001) decreases in colony formation ability in both cells depending on the dose increase (Figure 9a and b). When the same doses were compared in both cells, it was revealed that there was a greater effect at low doses (1.56 and 3.125 μM) in HCT-116 (Figure 9a1) cells and at higher dose (6.25 μM) in HT-29 (Figure 9b1) cells. Colony formation results of both cells support our viability results.

### 3.3 Molecular docking between 3-nitrophenyl chalcone derivative and target proteins

**Figure 10:**
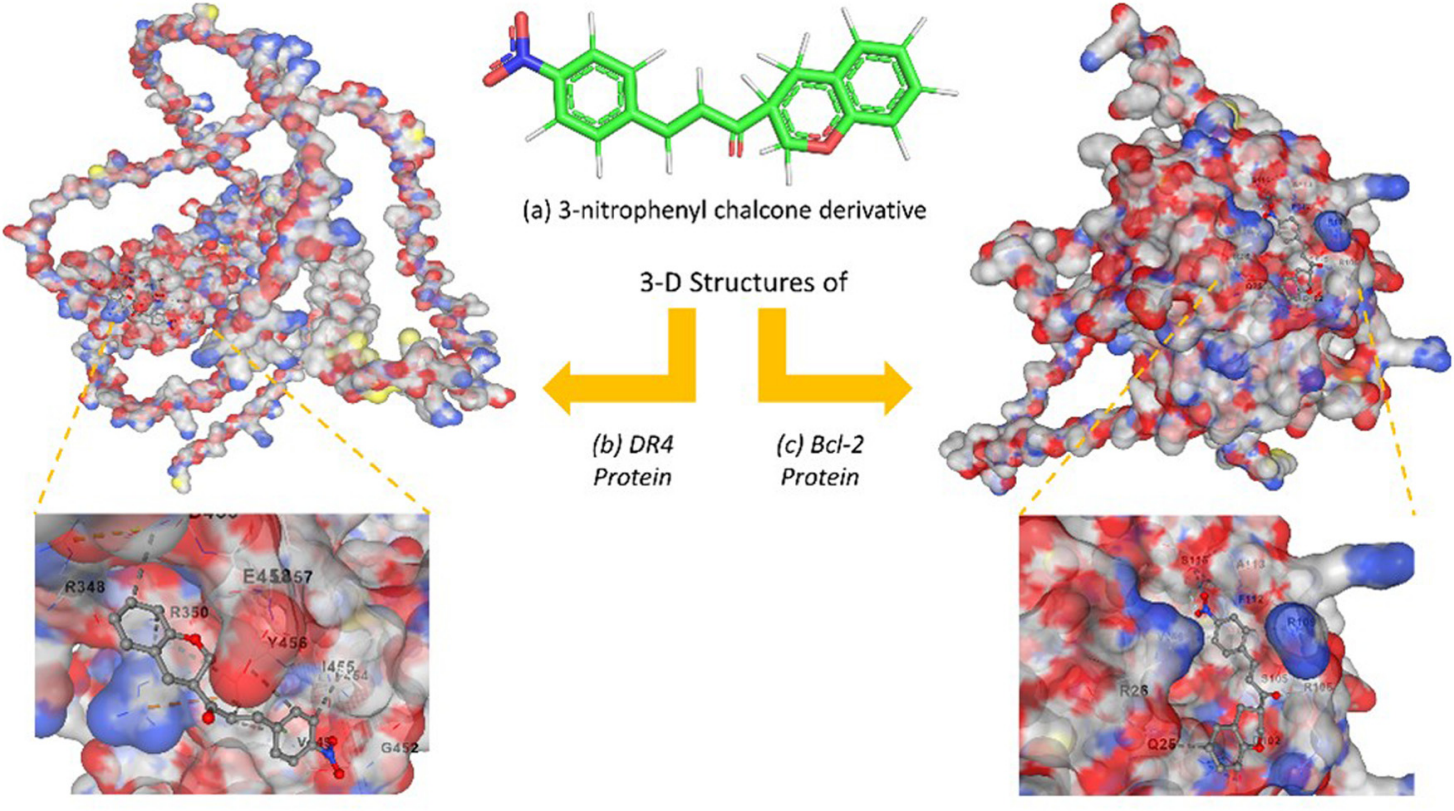
2D projection of the 3-nitrophenyl chalcone derivative (a); 3D-docked model of the derivative within the *DR-4* active site (b); and 3D-docked model of the derivative within the *BCL-2* active site (c).

Molecular docking was used to investigate the atomic-level interactions between 3-nitrophenyl chalcone derivative and target protein (*DR-4* and *BCL-2*). Three-dimensional structures of 3-nitrophenyl chalcone derivative and target protein are shown in Figure 10. According to the results, the binding scores of the target proteins *DR-4* and *BCL-2*, which may interact with the 3-nitrophenyl chalcone derivative, were determined to be (−6.0) and (−6.6) kcal/mol, respectively (Table 2). These values indicate that the chalcone derivative could potentially interact significantly with these proteins.

**Table 2:** CB-Dock2 results of the molecular docking between 3-nitrophenyl chalcone derivative and *DR-4/BCL-2* proteins

Cb-Dock2 data	CurPocket ID	Vina score	Cavity volume (Å3)	Center (*x*, *y*, *z*)	Docking size (*x*, *y*, *z*)	Contact residues (highest Vina score)
3-nitrophenyl chalcone and *DR-4* interaction	C4	**−6.0**	150	4, 3, 32	24, 24, 24	Chain A: GLN347 ARG348 ARG349 ARG350 LEU351 HIS426 LEU429 ASP430 ARG441 GLU442 GLN445 ASP446 VAL449 GLY452 PHE454 ILE455 TYR456 LEU457 GLU458 ASP459 THR461
	C5	−5.8	121	−5, 0, 18	24, 24, 24	
	C1	−5.7	709	10, 5, 25	24, 24, 24	
	C2	−5.7	278	−41, 28, 52	24, 24, 24	
	C3	−5.4	229	−51, 18, 11	24, 24, 24	
3-nitrophenyl chalcone and *BCL-2* interaction	C1	**−6.6**	432	6, 11, −2	24, 24, 24	Chain A: TYR21 LYS22 GLN25 ARG26 ASP102 SER105 ARG106 ARG109 ARG110 PHE112 ALA113 SER116 GLU152 GLY155 VAL156 VAL159 GLU160 ASN163
	C2	−6.6	289	−9, 11, 12	24, 24, 24	
	C5	−6.6	162	13, −2, 1	24, 24, 24	
	C3	−5.8	191	−6 −11 −11	24, 24, 24	
	C4	−5.8	167	12, −8, −11	24, 24, 24	

## 4 Discussion

Incidence and mortality in cancer continue to increase rapidly worldwide due to etiological factors [37]. Due to the development of multiple resistance to chemotherapeutic drugs, targeted treatment regimen research is gaining importance day by day [38]. Nowadays, a large number of anticancer agents obtained from various natural sources are used in the clinic. Chalcone derivatives belonging to the flavonoid family attract great attention due to their bioactive components in their side groups [39]. In the literature, many natural or synthetic chalcone derivatives have been shown to cause various biological activities, especially anticancer activity [40,41,42]. In addition, the anticancer properties of various chalcone derivatives have been investigated in many studies involving our group [43,44,45]. This study contains findings on the mechanistic effect of the synthesized and characterized 3-nitrophenyl chalcone derivative on colon cancer cells.

According to our SRB results, while there was no effect on healthy colon cells (CCD-18Co) at low doses as a result of both 24 h and 48 h of treatment, a decrease in viability was observed in cancer cells starting from low dose. This revealed that the derivative was specific to the cancer cell. The low IC₅₀ value indicates that the compound may exert its effect with minimal systemic toxicity, due to its efficacy at lower concentrations [46,47,48]. An *in vitro* study conducted on HCT-116 cells, the effect of quinazolinone chalcone derivative was investigated and IC_50_ values were calculated as 600, 140, 26, and 16 μM after 6, 12, 24, and 48 h, respectively [49]. In our study, as a result of the treatment applied to the same cells, it was observed that the cytotoxic effect was higher with IC_50_ values of 3.47 μM for 24 h and 1.7 μM for 48 h. This difference may have emerged thanks to 3-nitrophenyl ring in the chemical structure of our derivative. The low IC₅₀ value (∼1.71 µM) observed in HCT‑116 cells reveals that the derivative exhibits strong cytotoxic effects at low concentrations and is consistent with similar chalcone analogues in the literature, such as chalcone 6a which shows IC₅₀  =  2.37 ± 0.73 µM in HCT‑116 cells. It is estimated that this effect occurs due to the nitro group in the B ring and the acetyl group in the A ring increasing the electron-withdrawing capacity of the chalcone compound [50]. Additionally, in various studies, the SI of compounds with an SI value >3 was considered significant [51,52]. According to the index values obtained in this study, it is seen that there is a selective effect (⁓8) on HCT-116 cells in both times.

Apoptotic markers were analyzed to determine whether the observed decrease in cell viability was due to apoptosis-mediated cell death. Considering our triple fluorescent staining and caspase 3/7 flow cytometry results together, it was observed that late apoptosis pathway was active in the same dose groups in both cells at 24 h, and early apoptosis pathway was active at 48 h. In a recent study, the effects of Pd(ii) complex on breast cancer were investigated, and similar to our results, an increase in the early stage of apoptosis was observed in the transition from 24 to 48 h as a result of fluorescent staining [53]. This increase was thought to result from the upregulation of receptors involved in the extrinsic apoptosis pathway, such as phosphatidylserine. Similarly, according to our western blot results, the level of *DR-4* protein, which is involved in *TNFR1*-mediated apoptosis in the extrinsic pathway, increased dose-dependently after 48 h of treatment, supporting our idea. Additionally, the increase in *DR-4* levels and *caspase 3* activation according to flow cytometry results revealed that the *caspase 8* signaling cascade was stimulated by the extrinsic pathway [54,55]. Additionally, *PARP*, *c-PARP*, *BIP,* and *BCL-2* protein levels were investigated by western blot. It was observed that the level of *PARP* protein decreased and the level of the *c-PARP* subunit increased. Considering that *PARP* is the substrate of caspase 3 and 7 proteases and that a *c-PARP* subunit is formed as a result of enzymatic cleavage, a correlation was revealed in our western blot and flow cytometry results. Enzymatically active c-*PARP* plays a role in the process of apoptosis by irreversibly binding to the ends of DNA and causing DNA damage [56,57]. *BIP* protein ensures that proteins produced in the ribosome are folded correctly in the ER. One of the characteristics of cancer cells is the need for high protein synthesis to produce cellular metabolites [58,59]. ER-mediated apoptosis, which occurs through the *PERK/ATF4/IRE1* pathway due to the increase in the amount of *BIP* protein, which recognizes protein redox imbalance, is one of the main targets in treatment [59,60]. In this study, the dose-dependent increase in *BIP* level in both cells revealed that ER-mediated apoptosis was activated. *BCL-2* is an antiapoptotic protein that prevents the process of apoptosis by inhibiting the formation of the apoptosome complex. As a result of various 3D-QSAR, *in vitro* and *in vivo* studies, it has been observed that chalcone derivatives with anticancer potential initiate mitochondria-mediated caspase activation by suppressing *BCL-2* [61,62]. In line with findings from the literature, our results showed a dose-dependent reduction in *BCL-2* protein levels in colon cancer cells. When the molecular mechanisms of action of the *DR-4* and *BCL-2* pathways are examined together, molecular findings support the notion that the apoptosis-inducing potential of 3-nitrophenyl chalcone derivative occurs through both extrinsic and intrinsic apoptotic pathways (Figure 6). The IC_50_ values determined in HCT-116 and HT-29 cells and the increase in *DR-4* expression after 48 h of treatment are consistent with literature data indicating that chalcone derivatives increase sensitivity to TRAIL by increasing *DR-4/5* receptor levels on the cell membrane [63]. The fact that this effect is more pronounced in HCT-116 cells with an intact p53 gene is consistent with studies showing that p53 upregulates the response to TRAIL by increasing *DR-4* expression [64,65]. Moreover, the dose-dependent reduction in anti-apoptotic *BCL-2* expression supports the involvement of the intrinsic (mitochondria-mediated) apoptotic pathway. This finding is consistent with studies demonstrating that nitro-substituted chalcone derivatives disrupt mitochondrial membrane integrity and induce apoptosis by suppressing *BCL-2* expression. For example, in one study, a hybrid chalcone, chalcone 1C, decreased *BCL-2* protein levels in hepatocellular carcinoma cells. This resulted in disruption of mitochondrial membrane integrity and cytochrome c release. Apoptosome complex formation together with cytochrome c increased caspase 3 and 9 protein levels resulting in apoptotic responses in the nucleus. [66]. However, despite the more pronounced suppression of *BCL-2* levels in HT-29 cells, the earlier and more intense onset of apoptosis in HCT-116 cells (Figures 4 and 5) may be attributable to the presence of an activated p53 signaling pathway, which leads to increased *DR-4* expression.

The cell cycle is regulated by cyclin and *CDK* proteins depending on factors such as growth factors, cytokines, and oncogenes. It is known that uncontrolled cell division occurs in cancer cells as a result of the inhibition of cycle control [67]. In a study, 3, 4, 4, 5-tetramethoxy chalcone derivatives were found to cause an antiproliferative effect by suppressing division in colon and prostate cancer cells in the G1 and G2 stages, respectively [13]. Similarly, in this study, arrest in the G0/G1 phase was observed in both cancer cells as a result of treatment with 3-nitrophenyl chalcone derivative for 24 h. The presence of cells in this phase indicates that *CDKs* at the G1-S cycle checkpoint are activated. The treatment period was determined by taking into account the duration of each division cycle of HCT-116 and HT-29 cells as a result of literature analysis.

Another feature of cancer cells is that they gain the ability to metastasize and invade [68]. There are many studies in the literature using chalcone derivatives to reduce the migration and colony formation ability of cancer cells. For instance, it has been demonstrated that the L2H17 chalcone derivative inhibits the invasion and migration of mouse colon cancer (CT26.WT) cells [69]. In another recent study conducted on esophageal cancer cells, they observed that the derivative formed as a result of the hybridization of the heterocyclic chalcone skeleton and the dithiocarbamate compound slowed down growth depending on the dose and suppressed invasion and migration as a result of the decrease in *N-cadherin* and *Slug* biomarkers [70]. In this study, as a result of 48 h treatment with 3-nitrophenyl chalcone derivative, it was observed that the migration and colony formation abilities of colon cancer cells were suppressed dose-dependently. In addition, when the migration results in both cells were compared (Figure 8a and b), it was observed that the cells that lost their adhesion ability left from the plate surface, especially since the 12.5 uM dose showed a toxic effect on HCT-116 cells. In conclusion, the chalcone derivative showed stronger anticancer effects in HCT-116 cells, potentially due to their p53-positive genetic background, which enhances the suppression of cell growth and proliferation.

Recent advances in artificial intelligence have been playing an increasingly critical role in drug design and molecular modeling processes [71,72]. In this study, the anticancer potential of a 3-nitrophenyl chalcone derivative was evaluated using AI-assisted 3D modeling and molecular docking analyses. Binding affinity calculations performed with AutoDock Vina revealed that the compound exhibited binding energies in the range of –6 to –7 kcal/mol with its target proteins. These values are considered moderate-to-good binding affinities in the literature and indicate that the compound may form biologically meaningful interactions [73]. In particular, the observed binding score of –6.6 kcal/mol with the anti-apoptotic protein *BCL-2* suggests that the compound may have the potential to inhibit *BCL-2*. This finding is significant as it may contribute to the activation of apoptosis mechanisms in cancer cells. Overall, the docking results suggest that the 3-nitrophenyl chalcone derivative could be a promising multi-target anticancer drug candidate.

Based on the results of this study, it was revealed that 3-nitrophenyl chalcone derivative caused a strong cytotoxic effect on colon cells and death occurred by apoptosis in both cell lines (HCT-116 and HT-29). In the next investigation step, genes and proteins that are effective in metastasis, cell cycle, and life pathways will be determined through bioinformatic analysis. Thus, we aim to demonstrate the multifaceted biological effects of chalcone, which holds therapeutic potential in colon cancer.
